# Pediatric hematology/oncology healthcare professional emotional health during COVID‐19

**DOI:** 10.1002/cam4.4253

**Published:** 2021-09-01

**Authors:** Tal Schechter‐Finkelstein, Erin Plenert, Jennifer La Rosa, Jennifer McLean, K. Y. Chiang, Joerg Krueger, Erin Hearne, Lillian Sung

**Affiliations:** ^1^ Division of Haematology/Oncology The Hospital for Sick Children Toronto ON Canada; ^2^ Child Health Evaluative Sciences The Hospital for Sick Children Toronto ON Canada

**Keywords:** clinical guidelines, clinical management, pediatric cancer, psychosocial studies, quality of life, viral infection

## Abstract

**Objectives:**

Little is known about the impact of coronavirus disease 2019 (COVID‐19) on healthcare professional emotional health in pediatric hematology/oncology. Primary objective was to describe anxiety, depression, positive affect, and perceived stress among pediatric hematology/oncology healthcare professionals following a COVID‐19 outbreak. Secondary objectives were to compare these outcomes based on contact with a positive person, and to identify risk factors for worse outcomes.

**Materials and methods:**

We included 272 healthcare professionals working with pediatric hematology/oncology patients. We determined whether respondents had direct or indirect contact with a COVID‐19‐positive individual and then measured outcomes using the Patient‐Reported Outcomes Measurement Information System (PROMIS) depression, anxiety, and positive affect measures, and the Perceived Stress Scale.

**Results:**

Among eligible respondents, 205 agreed to participate (response rate 75%). Sixty‐nine (33.7%) had contact with a COVID‐19‐positive person. PROMIS anxiety, depression, and positive affect scores were similar to the general United States population. Those who had contact with a COVID‐19‐positive individual did not have significantly different outcomes. In multiple regression, non‐physicians had significantly increased anxiety (nurses: *p* = 0.013), depression (nurses: *p* = 0.002, pharmacists: *p* = 0.038, and other profession: *p* = 0.021), and perceived stress (nurses: *p* = 0.002 and other profession: *p* = 0.011) when compared to physicians.

**Conclusions:**

Pediatric hematology/oncology healthcare professionals had similar levels of anxiety, depression, and positive affect as the general population. Contact with a COVID‐19‐positive individual was not significantly associated with outcomes. Non‐physician healthcare professionals had more anxiety, depression, and perceived stress when compared to physicians. These findings may help to develop programs to support healthcare professional resilience.

## Graphical abstract

During a COVID‐19 outbreak, pediatric hematology/oncology healthcare professionals had similar levels of anxiety, depression, and positive affect compared to the general population. However, non‐physician healthcare professionals had more anxiety, depression, and perceived stress when compared to physicians.

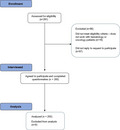

## 1 INTRODUCTION

The coronavirus disease 2019 (COVID‐19) pandemic has had a major impact on almost all individuals and healthcare systems globally. Healthcare professionals, particularly those with direct patient contact, have experienced particular challenges. Studies from previous infectious outbreaks have documented emotional stress with the potential for long‐lasting effects.^1^, ^2^ Early in the COVID‐19 pandemic, reports began to emerge describing the impacts on depression, anxiety, insomnia, and distress among healthcare professionals in China, Singapore, and around the globe.^3^, ^4^, ^5^ A major concern is the possibility of transmission from infected patients to healthcare professions, with the possibility of secondarily infecting their household contacts. Additional strains may be related to caring for acutely ill COVID‐19‐infected patients and resultant pressures on the healthcare system. The World Health Organization has mandated attention and intervention be focused on the increased emotional and physical burdens among healthcare professionals.^6^

With this emerging data about the impact of the COVID‐19 pandemic on healthcare professionals in general, it is important to consider whether the impact could be different within specific areas of medicine. One unique context is pediatric hematology/oncology. Within this setting, healthcare professionals are focused on a severely immunocompromised population with a high risk for treatment‐related mortality.^7^, ^8^, ^9^ It is possible that healthcare professionals working in pediatric hematology/oncology could have greater emotional distress related to the potential impact of COVID‐19 infection on these patients compared to general healthcare professionals. Understanding health outcomes and factors associated with them would help inform what programs are required to support healthcare professionals.

At our hospital, we experienced an outbreak of COVID‐19 on one of the pediatric hematology/oncology wards in April 2020, which led to infection of three healthcare professionals.^10^ This scenario meant that healthcare professionals not only had to cope with the COVID‐19 pandemic in the community but in addition, were faced with the potential for transmission in their work environment. Consequently, the primary objective was to describe anxiety, depression, positive affect, and perceived stress among pediatric hematology/oncology healthcare professionals following a COVID‐19 outbreak. Secondary objectives were to compare these outcomes based on contact with a positive person, and to identify risk factors for worse outcomes in the setting of the pandemic.

## 2 METHODS

This was a cross‐sectional, single‐center study that consisted of a quantitative and a qualitative component. This manuscript describes the quantitative component; the qualitative component will be presented elsewhere. The study was approved by the hospital research ethics board. Completion of the survey was considered implied consent to participate.

### 2.1 Eligibility criteria

We included physicians, nurses, pharmacists, social workers, child life specialists, dieticians, physiotherapists, and occupational therapists who worked on the hematology and oncology inpatient or outpatient units including the blood and marrow transplantation unit. We excluded those who did not work primarily with hematology/oncology patients, those who began to work on these units after the index case was diagnosed with COVID‐19, and those who had left the institution or who were on a leave of absence (including maternity leave) at the time of survey distribution.

### 2.2 Procedure

Eligible healthcare professionals were approached to participate by email. Those who agreed completed a demographic questionnaire. We asked whether they had contact with a person known to be infected with COVID‐19 including whether contact was direct physical contact or indirect (worked on the same ward/shift as a positive individual without direct contact). Next, they completed the outcome measures using REDCap.

### 2.3 Instruments

The instruments were the Patient‐Reported Outcomes Measurement Information System (PROMIS) anxiety, depression, and positive affect measures, and the Perceived Stress Scale.

The PROMIS anxiety item bank includes 29 items focusing on fear, anxious misery, hyperarousal, and somatic symptoms related to arousal (such as racing heart and dizziness). The PROMIS depression item bank contains 28 items specifically focused on negative mood, decrease in positive emotion, cognitive deficits, negative self‐image, and negative social cognition. The PROMIS positive affect item bank includes 34 items and it evaluates positive affective experiences such as feeling cheerful and attentive. For these PROMIS instruments, the mean and standard deviation of the United States general population (as determined pre‐pandemic) is 50 ± 10.^11^ A higher PROMIS score represents more of the concept being measured and thus direction does not consistently translate into better or worse health. Overall, results from large‐scale testing of PROMIS items in the general American population illustrate that all item banks demonstrate good reliability across most of the score distributions and construct validity was supported by moderate to strong correlations with legacy measures.^12^

The 10‐item Perceived Stress Scale (PSS‐10) is adapted from the original 14‐item scale; it examines respondents’ level of perceived stress over the past month. Rather than focusing on a single event, the PSS‐10 assesses the extent to which individuals feel unpredictable, uncontrollable, or overloaded in their lives.^13^ A total score ranging from 0 to 40 is calculated by reverse scoring the four positively worded items and then summing all the scale items. Higher scores are indicative of greater levels of perceived stress.^13^ The PSS‐10 demonstrates internal consistency reliability and convergent validity.^14^, ^15^

### 2.4 Potential risk factors

For regression analyses, risk factors examined were days from the index COVID‐19‐positive case, years treating pediatric cancer patients, profession type (physician, nurse, pharmacist, social worker, and other), trainee, male gender, contact with a COVID‐19‐positive individual, co‐habitation status (alone, roommate(s), family, or other), and whether co‐habitants have an underlying medical condition.

### 2.5 Statistical analysis

The primary aim was descriptive; PROMIS measures were considered similar to the general United States population if the median value fell within one standard deviation of the population mean. To compare outcomes between healthcare professionals who had contact with a COVID‐19‐positive person versus those who did not, and to compare those with direct and indirect contact, Wilcoxon rank sum test was used. For the secondary objectives, potential risk factors were evaluated using univariate linear regression. Factors significant at *p* < 0.1 were included in multivariable analyses as long as collinearity (Spearman *r* ≥ 0.6) was not present. Statistical significance was defined as *p* < 0.05. All analyses were performed using R studio version 3.6.1, The R Foundation for Statistical Computing.

## 3 RESULTS

**FIGURE 1:**
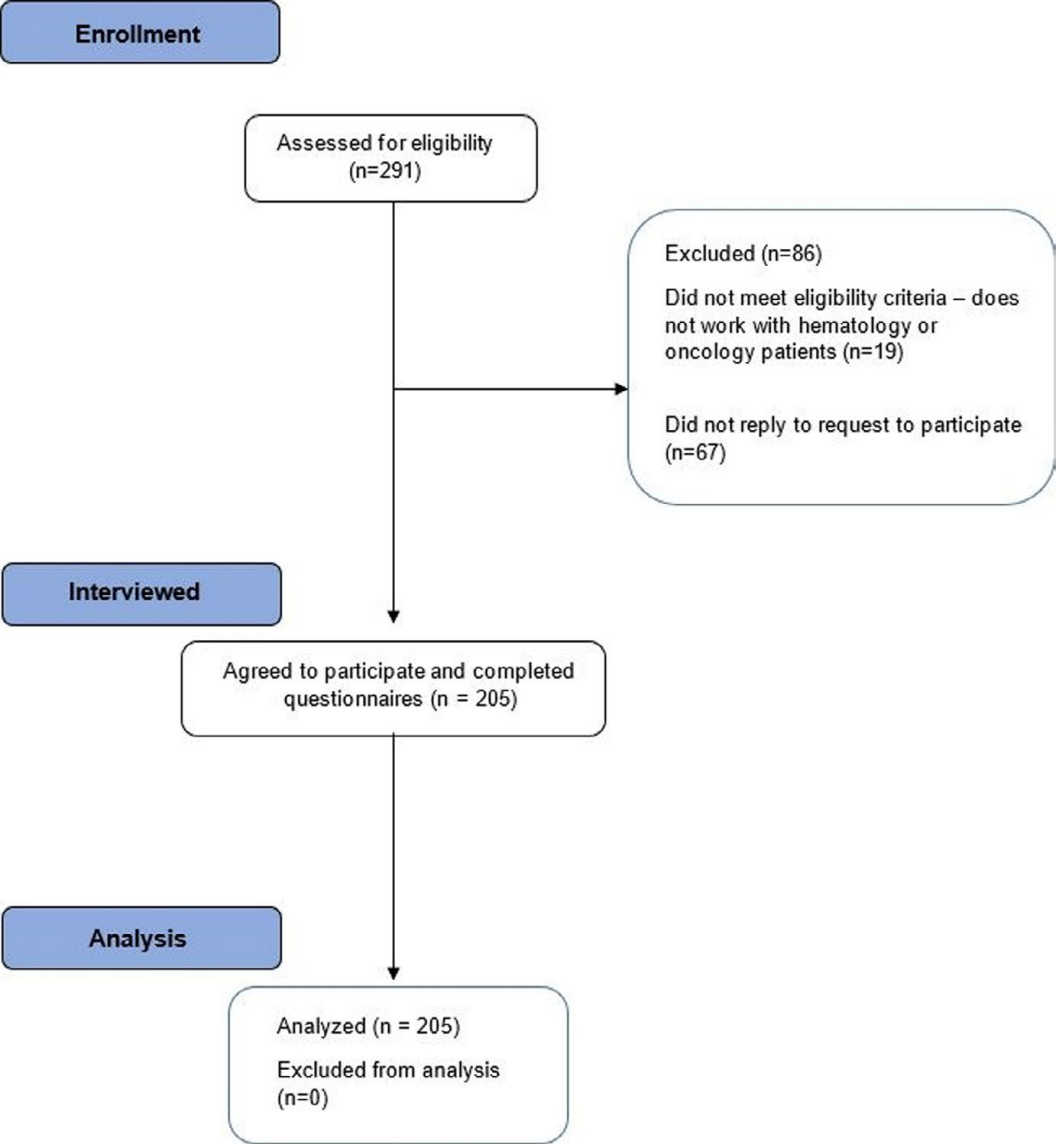
Flowchart of participant enrollment

Among the 272 eligible respondents, 205 pediatric hematology or oncology healthcare professionals participated in this study for a response rate of 75%. Of these, 122 (59.5%) were nurses, 56 (27.3%) were physicians, 8 (3.9%) were pharmacists, 6 (2.9%) were social workers, and 13 (6.3%) were other professional types including physiotherapy, occupational therapy, and child life specialists. Survey completion occurred between 9 June 2020 and 22 October 2020. Figure 1 shows the flow diagram of participant evaluation and reasons for exclusion. Table 1 illustrates demographics of the participant cohort. Sixty‐nine (33.7%) participants had contact with a COVID‐19‐positive individual; 43 had direct physical contact.

**TABLE 1:** Demographic characteristics of the cohort

	*N* = 205
Median days from index COVID−19 case (IQR)	175 (109–193)
Median years treating pediatric cancer patients (IQR)	11 (4–20)
Profession, *n* (%)	
Physician	56 (27.3)
Nurse	122 (59.5)
Pharmacist	8 (3.9)
Social worker	6 (2.9)
Other	13 (6.3)
Trainee, *n* (%)	27 (13.2)
Male, *n* (%)	38 (18.5)
Contact with COVID−19‐positive person, *n* (%)	69 (33.7)
Direct physical contact	43 (21.0)
Worked on same ward without direct contact	26 (12.7)
Co‐habitation status, *n* (%)	
Alone	18 (8.8)
Roommate(s)	11 (5.4)
Family	170 (82.9)
Other	6 (2.9)
Co‐habitants medical condition, *n* (%)	
Yes	44 (21.5)
No	143 (69.8)
NA	18 (8.8)

Abbreviations: COVID‐19, coronavirus disease 2019; IQR, interquartile range; NA, not available.

For the whole cohort, the median (interquartile range [IQR]) PROMIS T‐scores were as follows: anxiety 56.5 (52.0–59.8), depression 51.3 (46.1–55.0), and positive affect 45.0 (40.8–50.2). The median (IQR) of the Perceived Stress Scale was 17.^14^, ^15^, ^16^, ^17^, ^18^, ^19^, ^20^, ^21^, ^22^ The number of participants at high, moderate, and low stress were 9 (4.4%), 145 (70.7%), and 51 (24.9%), respectively.

Table 2 illustrates that those who had any contact (direct or indirect) with a COVID‐19‐positive individual did not have significantly different anxiety, depression, positive affect, or PSS‐10 scores compared to those who did not have contact with a positive individual. There was also no significant difference in scores between those who had direct and indirect contact (data not shown).

**TABLE 2:** Anxiety, depression, positive affect, and Perceived Stress Score by COVID‐19 contact

	No contact *n* = 136	Contact *n* = 69	*p* value*
Median PROMIS anxiety T score (IQR)	56.5 (51.4, 59.9)	57.7 (53.5, 59.8)	0.287
Median PROMIS depression T score (IQR)	51.3 (45.9, 55.2)	51.2 (46.1, 54.3)	0.937
Median PROMIS positive affect T score (IQR)	44.4 (40.3, 50.2)	46.2 (42.0, 50.2)	0.141
Median Perceived Stress Scale (IQR)	17.0 (13, 22.0)	18.0 (14.0, 21.0)	0.282

Abbreviations: COVID‐19, coronavirus disease 2019; IQR, interquartile range.

* *p* value by Wilcoxon rank sum test.

Table 3 identifies factors associated with anxiety, depression, positive affect, and perceived stress. In terms of anxiety, fewer number of years treating pediatric cancer patients, professional type, female gender, and cohabitation were significantly associated with more anxiety. Compared to physicians, nurses had more anxiety and compared to living alone, those with a roommate had more anxiety. In consideration of multiple regression, collinearity was not observed. Factors independently associated with more anxiety (beta ± standard error [SE]) were as follows: fewer years treating pediatric cancer patients (−0.12 ± 0.05, *p* = 0.029); and nurses compared to physician (3.90 ± 1.55, *p* = 0.013). In terms of depression, profession type and female gender were significantly associated with more depressive symptoms with nurses and pharmacists having more symptoms compared to physicians. In consideration of multiple regression, collinearity was not observed. Factors independently associated with more depression (beta ± SE) were as follows: nurses compared to physicians (4.75 ± 1.52, *p* = 0.002); pharmacists compared to physicians (5.60 ± 2.68, *p* = 0.038), and other profession compared to physicians (5.21 ± 2.25, *p* = 0.021). None of the measured variables were significantly associated with positive affect.

**TABLE 3:** Factors associated with anxiety, depression, positive affect, and perceived stress by linear regression

	Anxiety		Depression		Positive affect		Perceived Stress Scale	
	Beta ± SE	*p* value	Beta ± SE	*p* value	Beta ± SE	*p* value	Beta ± SE	*p* value
Days from index COVID−19 case	0.01 ± 0.01	0.223	0.01 ± 0.01	0.226	−0.00 ± 0.01	0.648	0.00 ± 0.00	0.953
Years treating pediatric cancer patients	−0.15 ± 0.05	0.003	−0.08 ± 0.05	0.131	−0.07 ± 0.05	0.206	−0.09 ± 0.04	0.030
Profession		0.0002		0.0007		0.276		0.034
Physician	REF		REF		REF		REF	
Nurse	4.80 ± 1.05	<0.0001	4.23 ± 1.03	<0.0001	−0.04 ± 1.11	0.974	2.18 ± 0.88	0.014
Pharmacist	3.21 ± 2.45	0.191	5.08 ± 2.40	0.036	−4.71 ± 2.59	0.071	1.04 ± 2.05	0.614
Social worker	3.144 + 2.79	0.261	0.05 ± 2.73	0.986	−1.58 ± 2.95	0.591	−1.13 ± 2.33	0.628
Other	5.77 ± 2.00	0.004	4.68 ± 1.96	0.018	−2.56 ± 2.11	0.227	4.11 ± 1.67	0.015
Trainee	−2.71 ± 1.39	0.053	−2.45 1.36	0.072	0.68 ± 1.42	0.632	−0.95 ± 1.14	0.404
Male	−3.97 ± 1.19	0.001	−2.92 ± 1.17	0.014	1.42 ± 1.24	0.253	−2.82 ± 0.97	0.004
Contact with COVID−19‐positive person	1.29 ± 1.00	0.199	0.40 ± 0.98	0.683	1.85 ± 1.01	0.069	1.06 ± 0.81	0.196
Co‐habitation status		0.010		0.333		0.448		0.109
Alone	REF		REF		REF		REF	
Roommate(s)	7.66 ± 2.54	0.003	3.52 ± 2.52	0.165	−4.03 ± 2.63	0.128	4.29 ± 2.10	0.042
Family	1.91 ± 1.65	0.248	−0.20 ± 1.63	0.901	−2.29 ± 1.71	0.181	1.22 ± 1.36	0.372
Other	5.90 ± 3.13	0.061	−1.01 ± 3.11	0.747	−2.63 ± 3.25	0.419	4.33 ± 2.58	0.095
Co‐habitants medical condition	0.24 ± 1.17	0.841	−0.16 ± 1.16	0.890	1.42 ± 1.18	0.233	0.83 ± 0.97	0.397

Abbreviations: COVID‐19, coronavirus disease 2019; REF, reference category.

In terms of the PSS‐10 scores, fewer number of years treating pediatric cancer patients, professional type, and female gender were significantly associated with more perceived stress. In consideration of multiple regression, collinearity was not observed. Factors independently associated with more perceived stress (beta ± SE) were as follows: nurses compared to physicians (4.90 ± 1.55, *p* = 0.002); and other profession compared to physicians (5.91 ± 2.29, *p* = 0.011).

## 4 DISCUSSION

In this study of pediatric hematology and oncology healthcare professionals conducted during the COVID‐19 pandemic, we found that when compared the United States general populations, anxiety, depression, and positive affect scores were similar. While we found that contact with a COVID‐19‐positive individual was not significantly associated with these outcomes, healthcare professional type was the most consistent associated risk factor. More specifically, non‐physician healthcare professionals had more anxiety (nurses), depression (nurses, pharmacists, and other professionals), and perceived stress (nurses and other professionals) when compared to physicians. Fewer years treating pediatric oncology patients was associated with more anxiety.

Our findings that anxiety, depression, positive affect, and perceived stress were not higher than population averages and were not associated with contact with a COVID‐19‐positive person are inconsistent with several studies. Lv et al. found that anxiety, depression, and sleep disorders were worse among healthcare professionals during the pandemic when compared to baseline values preceding the pandemic.^16^ In a rapid systematic review, Muller and colleagues identified 59 studies focused on mental health, the vast majority of which emanated from China.^17^ This review found that exposure to COVID‐19 was the most common factor associated with worse mental health. However, as most studies did not compare the results to baseline before the pandemic or to the general population, a causative relationship with the pandemic was unclear.^17^

In contrast, our results are concordant with a study conducted in Toronto after the 2003 outbreak of severe acute respiratory syndrome concluding that the incidence of new psychiatric disorders was similar to, or lower than community incidence rates. The authors suggested the results pointed to healthcare professional resilience.^2^ It is possible that anxiety, depression, positive affect, and perceived stress of pediatric hematology and oncology healthcare professionals were relatively favorable related to the low risk of COVID‐19 in pediatric patients in general,^18^ in pediatric hematology/oncology patients in particular,^19^, ^20^ or good outcomes among pediatric hematology/oncology infected patients.^19^, ^20^ It is also possible that the impact of the pandemic was lessened in pediatric hematology/oncology healthcare professionals given that they routinely work with severely ill pediatric patients with life‐threatening illness and thus, already have mechanisms established to maintain resilience.

While the PROMIS scores provide a comparison to the general population by their nature, the Perceived Stress Scale does not provide a similar comparative metric on its own. The median Perceived Stress Scale score in our study was 17, with about 75% of the participants reporting moderate or high stress levels. These scores are similar to the baseline scores found in a randomized controlled trial of a mindfulness‐based program among healthcare professionals in Bethesda, Maryland, where the mean score among the control group was 18.8 (standard deviation 6.4).^21^

We also found that non‐physicians had more anxiety, depression, and perceived stress when compared to physicians. This result may point to differences in a sense of control over the work environment. For example, physicians at the hospital are not typically deployed to different work areas such as the emergency room or intensive care unit. In contrast, such re‐deployment may be possible for nurses. Consistent with our findings, a report from Italy assessed mental health of healthcare professionals during the pandemic and found that anxiety and depression were significantly worse among nurses when compared to physicians.^22^ However, in contrast, a report from China found that physicians and non‐physicians did not have significantly different anxiety or depression during the COVID‐19 pandemic.^23^ Differences from our study may be related to cultural factors or related to how these outcomes were assessed. Nonetheless, these findings suggest that understanding sources of anxiety, depression, and perceived stress will be important to healthcare professional well‐being and understanding them from the perspective of non‐physicians will be particularly important.

Put together, our study is important as it focuses on a specific context, namely pediatric hematology/oncology. This restriction to a particular context illustrates how the impact of the COVID‐19 pandemic may differ between different healthcare professional groups. Our results provide insight into how programs focused on maintaining healthcare professional resilience may need to differ based upon healthcare context.

The strengths of our study include, first, the utilization of the PROMIS measure, which allows for a comparison again the United States general population. Second, we restricted this study to pediatric hematology and oncology healthcare professionals, thus providing confidence in our results’ generalizability to this population. Third, our response rate of 75% is favorable. However, our results must be interpreted in light of several limitations. First, this study represented a single cross‐sectional evaluation and thus, we do not know whether there have been temporal changes in these outcomes with different aspects of the COVID‐19 pandemic.^24^ Second, associations with outcomes were observed during the COVID‐19 pandemic; they may not be generalizable outside of a pandemic. Third, the study represents professionals at a single institution. Results in different institutions and countries could differ.

## 5 CONCLUSION

In conclusion, pediatric hematology and oncology healthcare professionals had similar levels of anxiety, depression, and positive affect as the general population. Contact with a COVID‐19‐positive individual was not significantly associated with outcomes. Non‐physician healthcare professionals had more anxiety, depression, and perceived stress when compared to physicians. These findings may help to develop programs to support healthcare professional resilience during a pandemic.

## CONFLICT OF INTEREST

The authors have no conflict of interest relevant to this article to disclose.

## ETHICAL APPROVAL STATEMENT

The study was approved by the SickKids research ethics board. Completion of the survey was considered implied consent to participate.

## Data Availability

The datasets used or analyzed during this study are available from the corresponding author upon reasonable request.
